# 561. Accuracy of Burn-trained Physicians vs. Burn-specific Equation vs. Indirect Calorimetry in Predicting Energy Requirements

**DOI:** 10.1093/jbcr/irag033.301

**Published:** 2026-04-06

**Authors:** Briana Sylvester, Alexis Kimball, Anastasiya Ivanko, M Victoria P Miles, Jeffrey E Carter, Jonathan E Schoen

**Affiliations:** Louisiana State University Health Burn Center, Lansdale, PA; Burn Center at University Medical Center, Metairie, LA; University Medical Center Burn Center, Louisiana State University Health Sciences Center, New Orleans, New Orleans, LA; University Medical Center Burn Center, Louisiana State University Health Sciences Center, Department of Surgery, New Orleans, New Orleans, LA; University Medical Center Burn Center, Louisiana State University Health Sciences Center New Orleans, New Orleans, LA; UCI Health Regional Burn Center, New Orleans, LA

## Abstract

**Introduction:**

Severe burn injuries provoke a hypermetabolic response marked by elevated resting energy expenditure (REE), catabolism, and immune dysregulation. Proper nutritional support is critical in burn care, yet estimating caloric needs remains challenging. While burn-specific equations and physician judgment are commonly used to guide nutrition, indirect calorimetry is considered the gold standard per ASPEN guidelines for determining true metabolic demand. Here, we evaluate caloric need estimations from burn attendings and residents and the Curreri formula in relation to REE provided by indirect calorimetry.

**Methods:**

A prospective analysis was conducted on burn patients (3-58% Total Burn Surface Area (TBSA)) from July 2024 to July 2025 at a single, ABA-verified burn center. Estimates of caloric needs from burn attendings (who have completed a burn and critical care fellowship), residents (general surgery, post-graduate years 1-2) and Curreri calculation were collected for comparison to the REE obtained by indirect calorimetry (IC). A total of 61 REE estimations were obtained; 30 from burn attendings and 31 from general surgery residents. The primary outcome evaluated was the degree of deviation from measured REE.

**Results:**

Nineteen patients were included in the analysis. Preliminary estimates by attending physicians were found to differ significantly from values obtained by indirect calorimetry, with an average deviation from actual REE by 21.3% (absolute value) and ranging from -66% of actual REE to +45% of actual REE. For attending physicians, 9 of 32 estimations were within ±10% of the indirect calorimetry REE. Resident physician estimations averaged 19.2% off from measured REE (absolute value), with a range of -56% of actual to +65% of actual. For resident physicians, 14 out of the 32 estimations were within ±10% of the metabolic cart REE. REE was multiplied by a 1.3 activity factor to get total daily calories and was compared to the Curreri formula with a range of -37% of actual to +38% of actual. It was noted that 12 out of the 32 Curreri estimations were within ±10% of the indirect calorimetry REE with an activity factor of 1.3. Across the cohort, indirect calorimetry revealed a wide range of actual REE values, underscoring the limitations of generalized estimation methods in hypermetabolic burn patients.

**Conclusions:**

Burn-induced hypermetabolism remains a complex, multifactorial challenge. Serial indirect calorimetry provides an objective, non-invasive, and cost-effective tool to guide accurate nutritional needs, minimize complications such as excessive weight loss or gain, and enhance clinical decision-making throughout the continuum of burn care.

**Applicability of Research to Practice:**

These findings support the integration of indirect calorimetry into routine burn care to provide personalized and accurate nutritional support, potentially improving patient outcomes.

**Funding for the study:**

N/A.

